# Effect of graded levels of soapnut (*Sapindus mukorossi*) shell powder on reproductive performance in broiler breeders

**DOI:** 10.5713/ajas.18.0353

**Published:** 2018-07-26

**Authors:** S. K. Chaudhary, A. B. Mandal, R. Bhar, M. Gopi, A. Kannan, S. E. Jadhav, J. J. Rokade

**Affiliations:** 1 Animal Nutrition Division, ICAR-Indian Veterinary Research Institute, Izatnagar, Bareilly, Uttar Pradesh 243122, India; 2 Acting Director, ICAR-Central Avian Research Institute, Izatnagar, Bareilly, Uttar Pradesh 243122, India; 3 Animal Nutrition Lab, ICAR-Indian Veterinary Research Institute, Eastern Regional Station, Kolkata, West Bengal 700037, India; 4 Avian Physiology and Reproduction Division, ICAR-Central Avian Research Institute, Izatnagar, Bareilly, Uttar Pradesh 243122, India; 5 Animal Nutrition Lab, ICAR-Directorate of Poultry Research, Rajendra Nagar, Hyderabad, Telangana 500030, India; 6 Avian Genetics and Breeding Division, ICAR-Central Avian Research Institute, Izatnagar, Bareilly, Uttar Pradesh 243122, India

**Keywords:** Soapnut, Sapindus, Saponin, Fertility, Hatchability, Broiler Breeders

## Abstract

**Objective:**

This study investigated the effects of soapnut (*Sapindus mukorossi*) shell powder (SSP) on serum hormone level, egg quality, semen characteristics and reproductive performance of broiler breeders fed with a maize-soybean meal based diet.

**Methods:**

Ninety six female and twenty four male CARIBRO-VISHAL broiler breeders, 38-week old, were individually caged and randomly allocated to four treatment groups (24 female breeders/treatment and 6 male breeders/treatment): an un-supplemented control (T1) and three groups with 0.0176% SSP (group T2), 0.026% SSP (group T3) and 0.0528% SSP (group T4), to have supplementary saponin at 0, 50, 75, and 150 ppm, respectively, for 42 days.

**Results:**

The results indicated that serum (p<0.001) and seminal plasma (p<0.05) testosterone level, semen volume (p<0.001), mass motility (p<0.001), and live spermatozoa count (p<0.001) was increased in groups T3 and T4 compared to T2 and control groups. Compared with control group, total sperm count was increased (p<0.001) and dead spermatozoa count was decreased (p<0.001) in SSP supplemented groups. Supplementation of SSP did not affected the quality of egg lay. Compared with control group, fertility (p<0.01) and hatchability (total eggs set and fertile eggs set) (p<0.001) were significantly improved in SSP supplemented groups with the highest improvement in T3 treatment group. Embryonic death was decreased (p< 0.001) in SSP supplemented groups compared to control; lowest embryonic death was recorded in T3 treatment group.

**Conclusion:**

Thus, it was concluded that dietary supplementation of 0.026% SSP (saponin equivalent 75 ppm) improved the reproductive performance of broiler breeders.

## INTRODUCTION

The poultry industry is one of the fastest growing animal enterprises worldwide. High-quality nutrition is crucial for breeders to transfer fertile and hatchable eggs through constant and well-balanced nutritional supply for the normal development of the embryos [1]. Embryonic as well as early post-hatch period represents about 85% of entire lifespan that is critical for attaining quality broilers at marketing [2–4]. Moreover, the broiler breeders remain under constant stress during their production cycle due to higher lay and handling for vaccination, semen collection and insemination. Climate is the other factor which is responsible for stress in tropical countries like India where hot-dry and hot-humid weather covers for more than seven months of the year. Stress not only curtails production of the birds but also imparts welfare issues, like, enhanced mortality rate, discomfort and disease [5]. Thus, to conquer these main challenges, it is requisite to develop some extraneous approach.

*Sapindus mukorossi* (soapnut) found over most of the hilly region of Garhwal Himalaya of India is one of the most important members of Sapindaceae family. The fruit pericarp has been used traditionally as a medicine in Indian Ayurveda since decades. It is considered to be a rich source of saponins (high molecular weight plant secondary metabolites), which exhibits several biological and pharmacological activities such as antihyperlipidemic, antioxidant, anti-inflammatory [6], tyrosinase inhibition [7], antifungal, hepatoprotective and adjuvant activities [8,9]. Due to its various activities it is expected to improve the performance under stress condition.

Supplementation of saponin results in an increased seminiferous tubule diameter and testis size in male broiler birds [10]. Studies in male rats [11,12] also revealed an improvement in sperm quality along with higher testosterone concentration following saponin administration. The similarity in chemical structure of saponins to steroid hormones, could influence their levels and activity. Several researchers have been reported that Yucca supplementation to layer diet had no effect on egg production and its quality but reduced number of cracked eggs [13]. However, the effect of saponin supplementation on fertility and hatchability is not very clear and reported to exert variable results. Studies on the effect of saponin containing soapnut shell powder (SSP) at the different level of incorporation, on hormonal profile, egg quality, semen characteristics, fertility, hatchability and embryonic mortality in broiler breeders are very limited. Thus, the present study was aimed to investigate the effect of different dietary levels of SSP on reproductive performance in broiler breeders.

## MATERIALS AND METHODS

The experiment was carried out at the experimental broiler farm, Central Avian Research Institute Izatnagar, India and was approved by the Institute Animal Ethics Committee (IAEC).

### Preparation of soapnut shell powder

Soapnut fruits were purchased from local market of Palampur, District-Kangra, Himachal Pradesh, India. Seeds were separated manually from the dried fruits. The collected soapnut shells were dried at room temperature and grind in the mixer to a uniform particle size and stored in an air tight container for the further experimental purpose. Saponin was estimated from SSP as per the method described by Sharma et al [14] and expressed on percent dry matter basis (DMB). The saponin yield from the sample was 28.4% DMB. Total phenol, non tannin phenol, total tannin, condensed tannin, and hydrolysable tannin in SSP was found to be 1.43%, 0.60%, 0.83%, 0.36%, and 0.47%, respectively.

### Experimental birds and design

About thirty-eight week age of ninety-six female and twenty-four male broiler breeders of CARIBRO-VISHAL breed were selected for the study. The birds were randomly assigned to four treatment groups: T1 (control), T2, T3, and T4 consisting of 24 female and 6 male breeders in each. Both female and male broiler breeders were reared separately. Duration of the experiment was of 42 days (6 weeks). All the birds were housed in battery cages with uniform and standard managemental condition. The mean maximum and minimum temperature was 39°C and 21°C, respectively. The relative humidity during the study period was between 45% to 50%. Birds were provided 16 h lighting. The experimental diets contained 0, 0.0176%, 0.026%, and 0.0528% SSP (dose equivalent: 0, 50, 75, and 150 ppm saponin), respectively. The levels of saponin were based on the findings of Miah et al [10]. The ingredients and nutrient composition of basal diet [15] is presented in Table 1.

### Assessment of serum hormone level

Blood samples were collected from the jugular vein of six female and six male breeders per treatment at fortnight interval, before feeding and watering, in commercially available clot activating tubes for serum. Serum samples were analyzed for estrogen and testosterone hormone using ELISA Kits purchased from Labor Diagnostika Nord (LDN), Nordhorn, Germany.

### Determination of egg quality

Thirty-two eggs (eight eggs per treatment) from their respective treatment groups were collected at fortnight interval to assess the quality of eggs lay. External egg quality was examined for egg weight, shape index (maximum breadth of egg in mm/maximum length of egg in mm), shell color and shell thickness, whereas, internal egg quality was examined for albumen index (average albumen height in mm/ average albumen width in mm), Haugh unit, yolk index (yolk height in mm/yolk width in mm) and yolk color.

### Determination of semen quality

Semen samples from the six male broiler breeders per treatment were collected at fortnight interval, before feeding and watering by the abdominal massage method [16]. Semen samples were examined for their physical characteristics such as semen volume, color, total sperm count, mass motility, live spermatozoa count, and dead spermatozoa count. Seminal plasma was harvested to examine its biochemical properties. For this, the collected fresh semen samples were centrifuged at 4°C in a refrigerated centrifuge at 5,000 rpm for 10 minutes. The resulting supernatant was transferred into a clean polypropylene tube using Pasteur pipette and stored under cold conditions (−20°C) till further analysis. These samples were analyzed for biochemical parameters (glucose, total protein, uric acid, total cholesterol, calcium, inorganic-phosphorus, aspartate transaminase (AST) and alanine transaminase (ALT) using diagnostic kits (purchased from Coral Clinical System, Goa, India) along with testosterone level using ELISA Kit (purchased from Labor Diagnostika Nord, Germany).

### Artificial insemination and determination of fertility and hatchability

After 42 days of feeding trial the birds were assessed for their fertility and hatchability performance. Semen samples were pooled from their respective treatment male birds and artificial insemination (AI) was done in their respective treatment female birds. The eggs were collected up to 5–7 days post-AI and transferred to the hatchery for the evaluation of fertility, hatchability and embryonic mortality.

### Statistical analysis

The data generated from the experimental study were subjected to statistical analysis using One-way analysis of variance procedure of SPSS 20.0 computer software. Duncan multiple range test [17] was used for verifying significant difference among treatment means and p<0.05 was considered to be statistically significant.

## RESULTS

### Effect on hormonal profile

The serum and seminal plasma testosterone assay were performed for male, whereas, serum estrogen assay was performed for female broiler breeders which are presented in Table 2. The treatment mean for serum testosterone (ng/mL) was significantly (p<0.001) higher in T3 (4.56) and T4 (5.64) groups compared to control (3.63), whereas, T2 (4.38) group shown an intermediate response. The treatment mean for seminal plasma testosterone (ng/mL) was significantly (p<0.05) higher for T4 (1.65) group compared to T1 (1.41) and T2 (1.47) group, whereas, T3 (1.55) group shown an intermediate response. The treatment mean for serum estrogen (pg/mL) was comparable (p>0.05) among the groups.

### Effect on egg quality

Table 3 shows the effects of SSP on the quality of eggs produced. The treatments had no significant (p>0.05) effects on egg weight (g), shape index (%), shell color, shell thickness (10^−2^ mm), albumin index (%), Haugh unit, yolk index (%), and yolk color.

### Effect on semen quality

**Figure 1:**
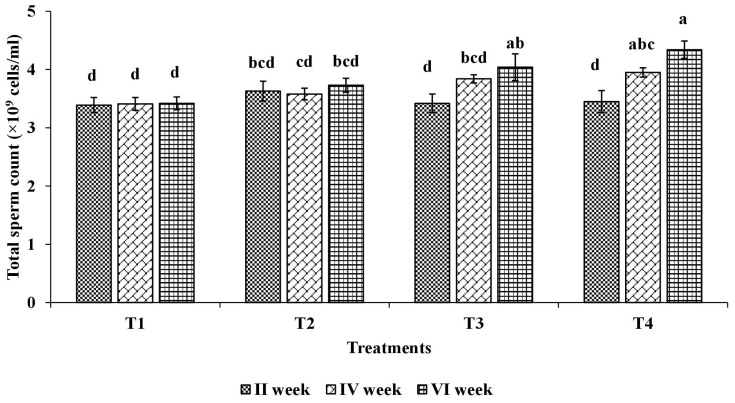
Effect of soapnut shell powder (SSP) supplementation on total sperm count in male broiler breeders (T×P interaction). T1, control group; T2, control+SSP at 0.0176%; T3, control+SSP at 0.026%; T4, control+SSP at 0.0528%. Bars with different superscripts differ significantly (p<0.05).

The effect of SSP on semen physical characteristics for different dietary treatment groups are presented in Table 4. Compared with the control group and group T2, the semen volume (mL) of group T4 and T3 was significantly (p<0.001) increased. The dietary SSP did not alter the milky white color of semen. Total sperm count (×10^9^ cells/mL) was significantly (p<0.001) increased in group T4 compared with groups T1 and T2, whereas, group T3 had shown an intermediate response (Figure 1). Compared with the control group and group T2, the mass motility (%) and live spermatozoa counts (%) were significantly (p<0.001) higher for groups T4 and T3. Furthermore, the dead spermatozoa counts (%) were significantly (p<0.001) decreased in groups T3 and T4 compared to control group and group T2. The analyzed biochemical constituents of seminal plasma are presented in Table 5. This was analyzed for glucose (mg/dL), total protein (g/dL), uric acid (mg/dL), total cholesterol (mg/dL), calcium (mg/dL), inorganic-phosphorus (mg/dL) AST (IU/L), and ALT (IU/L), which were found to be non-significant (p>0.05) among the treatments.

### Effect on fertility, hatchability, and embryonic mortality

Data related to fertility, hatchability and embryonic mortality are given in Table 6. Compared with control group, the fertility (%) and hatchability (%) of SSP treated groups were significantly increased with a highest increment in group T3. Furthermore, the embryonic mortality (%) was significantly (p<0.001) reduced in SSP treated groups compared to control group with a lowest mortality in group T3.

## DISCUSSION

### Effect on hormonal profile

Present experiment revealed that the serum and seminal plasma testosterone levels were significantly increased in male birds following SSP supplementation. In accordance with the present study, Zhan [18] noted that dietary incorporation of Camellia seed saponin had increased the serum testosterone level by 25.61% in broilers. Also according to Fahim et al [19], dietary supplementation of saponin rich *Panax ginseng* to the rats for 60 days had significantly increased the blood testosterone levels. *Panax ginseng* contains ginsenosides Rg1 which is a triterpenoid saponin and structurally resembles with the steroid hormones; are responsible for the increase in the level of serum testosterone and copulatory behavior of rats [11]. In studies of Hemalatha and Hari [12], the saponin rich fraction of *Tribulus terrestris* (SFTT) treatment caused a significant increase in the serum testosterone level in male rats. American ginseng contains ginsenoside Rb1; responsible for an increases secretion of luteinizing hormone by acting directly on the anterior pituitary gland [20]. According to Francis et al [21], Quillaja saponin was associated to stimulate the production of the luteinizing hormone in fish.

### Effect on egg quality

In the present study, dietary supplementation of SSP had no significant effects on the egg quality of broiler breeders. This is in accordance with Guclu [22] and Ayasan et al [23], reporting that dietary Yucca supplementation had no significant effect on shape index, albumin index, yolk index, Haugh unit and shell thickness of laying quails. Also according to Kutlu et al [24] and Alagawany et al [13], dietary Yucca supplementation had no significant effect on egg production, shell thickness, albumin and yolk weights as well as shape index but reduced number of cracked egg in layers. The different dietary levels of alfalfa meal had no significant effect on egg production, weight, mass, and shell breaking strength in quails [25]. Contrary to this, Afrose et al [26] reported that Karaya saponin had increased the egg weight, Haugh units and yolk index in laying hens.

### Effect on semen quality

Dietary SSP supplementation had significantly improved physical characteristic of semen, whereas, the biochemical characteristic were found to be similar among the different treatment groups. Several reports indicated that saponin from various sources could improve semen quality. Miah et al [10] reported that dietary supplementation of saponin resulted in an increased testis size in male broiler birds. Furthermore, Hong et al [27] had also reported an increased seminiferous tubule diameter in cockerels following dietary incorporation of saponin. Our results also correlate with previous findings of Hemalatha and Hari [12], as they reported that the SFTT treatment caused significant increase in sperm motility, sperm count, sperm morphology as well as testicular weight compare to non-treated controls male rats and hypothesized that SFTT promoted testicular development, therefore, enhances sperm production. In studies of Oyeyemi et al [28], increased sperm motility and concentration with increasing dose of saponin from *Vernonia amygdalina* treated groups resulted in an increased fertilizing capacity of the spermatozoa in male wistar rats. Ginseng-treated rats had demonstrated an increased rate of spermatogenesis via elevated expression of glial cell-derived neurotrophic factor in sertoli cells [29] as well as activation of testicular cAMP-responsive element modulator activity [30]. Also in an experiment of Balazi et al [31], *Yucca schidigera* administration increased the spermatozoa concentration and motility of rabbit buck.

### Effect on fertility, hatchability, and embryonic mortality

Present study indicated that fertility and hatchability was significantly improved, whereas, embryonic mortality was significantly decreased following dietary supplementation of SSP. Natural antioxidants such as, vitamin E, selenium, carotenoids, and various plant extracts can play an important role in avian reproduction by maintaining antioxidant defenses of the spermatozoa and embryonic tissues [32]. Therefore, an optimum dietary level of antioxidant supplement is necessary to maintain high productive and reproductive performances of commercial poultry. The increased fertility and hatchability in our study might be due to the antioxidant effect [6] of soapnut fruit. Improved fertility and hatchability due to saponin supplementation was also reported by Enaiat et al [33] who attributed their antioxidant property for the effect. The authors further credited the ability of saponin to reduce the ammonia concentration inside the shed which in turn improved the internal egg qualities (egg pH and albumin content). According to Ayasan [34], compared with control group dietary *Yucca schidigera* supplementation increased the fertility percent numerically without changing hatchability percent in quails. Also Olgun and Yildiz [25], reported that the different dietary levels of alfalfa meal had numerically higher fertility, hatchability of total eggs set or hatchability of fertile eggs in quails. Cline et al [35] found that feeding with Yucca extract to sows prior to furrowing resulted in a significant decrease in stillbirths cases. It was later confirmed by Herpin et al [36] that inclusion of whole Yucca powder in sow diets caused a reduction in stillbirths and increased viability of neonatal pigs. Also in an experiment of Stochmlova et al [37], *Yucca schidigera* administration increased release of ovarian progesterone, conception rate and kindling rate of rabbit does. More Recently, Foldesiova et al [38] confirmed an increased performance of rabbit does in terms of growth and fecundity following dietary supplementation of *Yucca schidigera* extract.

## CONCLUSION

From the present study, it is clear that dietary SSP supplementation had no effect on quality of egg lay, whereas, SSP at 0.026% significantly improved serum and seminal plasma testosterone level, semen characteristics, fertility and hatchability as well as significantly decreased embryonic mortality in broiler breeders. Thus, it can be concluded that, dietary supplementation of 0.026% SSP (saponin equivalent 75 ppm) improved the reproductive performance of broiler breeders.

## Figures and Tables

**Table 1:** Ingredients and nutrient composition of basal diet (DM basis)

Item	Percentage	

	Female	Male
Ingredients		
Maize	58.42	57.28
Soybean meal (44% CP)	28.90	15.60
Deoiled rice bran	2.10	23.00
Oyster shell	6.20	0.00
Calcite	2.00	1.80
Dicalcium phosphate	1.50	1.40
Trace mineral premix	0.15	0.15
DL-methionine	0.148	0.12
L-lysine	0.054	0.13
Common salt	0.259	0.263
Vitamin premix	0.15	0.15
B-complex	0.015	0.015
Choline chloride	0.05	0.05
Toxin binder	0.05	0.05
Coccidiostat	0.005	0.005
Nutrient composition		
CP1) (%)	16.0	15.0
ME2) (kcal/kg)	2800	2750
Ca1) (%)	3.50	1.00
Total P1) (%)	0.70	0.70
Available P2) (%)	0.44	0.44
Digestible Lys2) (%)	0.72	0.68
Digestible Meth2) (%)	0.40	0.35
Digestible Met+Cys2) (%)	0.66	0.62

DM, dry matter; CP, crude protein; ME, metabolizable energy; Ca, calcium; P, phosphorus; Lys, lysine; Met, methionine; Cys, cystine.

1) Means analyzed values.

2) Means calculated values.

**Table 2:** Effect of soapnut shell powder supplementation on serum hormones of broiler breeders

Attributes	Treatment groups1)				SEM	p-value		
	
	T1	T2	T3	T4		T	P	T×P
Serum testosterone in males (ng/mL)	3.63^c^	4.38^bc^	4.56^b^	5.64^a^	0.25	<0.001	0.382	0.923
Seminal plasma testosterone (ng/mL)	1.41^b^	1.47^b^	1.55^ab^	1.65^a^	0.05	<0.05	0.340	0.945
Serum estrogen in females (pg/mL)	190.91	190.94	194.48	194.38	3.50	0.820	0.942	0.635

SEM, standard error of the mean; T, treatment; P, period; T×P, interaction; SSP, soapnut shell powder.

1) T1, control group; T2, control+SSP at 0.0176%; T3, control+SSP at 0.026%; T4, control+SSP at 0.0528%.

a–c Means within a row with no common superscripts differ significantly (p<0.05).

**Table 3:** Effect of soapnut shell powder supplementation on egg quality of broiler breeders

Attributes	Treatment groups1)				SEM	p-value		
	
	T1	T2	T3	T4		T	P	T×P
Egg weight (g)	60.42	61.49	62.41	61.51	0.69	0.295	0.618	0.974
Shape index (%)	71.63	71.18	72.34	73.11	0.61	0.136	0.745	0.246
Shell colour	4.00	4.00	4.06	4.28	0.25	0.860	0.875	0.986
Shell thickness (×10^−2^ mm)	37.69	37.83	37.80	38.05	0.31	0.886	0.681	0.917
Albumin index (%)	12.76	12.71	12.96	13.08	0.34	0.850	0.224	0.755
Haugh unit	96.29	95.99	96.37	96.59	0.80	0.964	0.408	0.906
Yolk index (%)	50.96	50.26	51.48	51.89	0.64	0.299	0.632	0.194
Yolk colour	8.22	8.50	8.33	8.44	0.24	0.870	0.473	0.985

SEM, standard error of the mean; T, treatment; P, period; T×P, interaction.

1) T1, control group; T2, control+SSP at 0.0176%; T3, control+SSP at 0.026%; T4, control+SSP at 0.0528%.

**Table 4:** Effect of soapnut shell powder supplementation on physical characteristics of semen

Attributes	Treatment groups1)				SEM	p-value		
	
	T1	T2	T3	T4		T	P	T×P
Volume (mL)	0.62^c^	0.62^c^	0.74^b^	0.87^a^	0.04	<0.001	0.157	0.250
Total sperm count (×10^9^ cells/mL)	3.40^c^	3.65^b^	3.77^ab^	3.91^a^	0.09	<0.001	<0.01	<0.05
Mass motility (%)	83.67^b^	84.67^b^	86.93^a^	88.60^a^	0.83	<0.001	<0.001	0.302
Live spermatozoa count (%)	81.68^c^	83.12^b^	85.37^a^	86.41^a^	0.51	<0.001	<0.001	0.127
Dead spermatozoa count (%)	18.32^a^	16.88^b^	14.63^c^	13.59^c^	0.51	<0.001	<0.001	0.127

SEM, standard error of the mean; T, treatment; P, period; T×P, interaction.

1) T1, control group; T2, control+SSP at 0.0176%; T3, control+SSP at 0.026%; T4, control+SSP at 0.0528%.

a–c Means within a row with no common superscripts differ significantly (p<0.05).

**Table 5:** Effect of soapnut shell powder supplementation on biochemical characteristics of semen

Attributes	Treatment groups1)				SEM	p-value		
	
	T1	T2	T3	T4		T	P	T×P
Glucose (mg/dL)	52.50	52.59	52.73	52.38	0.39	0.938	0.433	0.630
Total protein (g/dL)	3.50	3.60	3.68	3.50	0.11	0.652	0.653	0.996
Uric acid (mg/dL)	7.84	7.66	7.47	7.97	0.20	0.328	0.266	0.932
Total cholesterol (mg/dL)	58.62	58.92	57.84	58.24	0.89	0.868	0.927	0.980
Calcium (mg/dL)	9.46	9.79	9.54	9.70	0.20	0.677	0.616	0.873
Inorganic-phosphorus (mg/dL)	5.31	5.30	5.31	5.32	0.08	0.997	0.999	1.000
ALT (IU/L)	13.25	13.25	13.08	13.17	0.27	0.974	0.837	0.987
AST (IU/L)	157.33	156.92	156.50	156.33	1.35	0.965	0.858	0.872

SEM, standard error of the mean; T, treatment; P, period; T×P, interaction; ALT, alanine transaminase; AST, aspartate transaminase.

1) T1, control group; T2, control+SSP at 0.0176%; T3, control+SSP at 0.026%; T4, control+SSP at 0.0528%.

**Table 6:** Effect of soapnut shell powder supplementation on fertility, hatchability and embryonic mortality among different dietary treatment groups

Treatment groups1)	Fertility (%)	Hatchability (TES, %)	Hatchability (FES, %)	Embryonic mortality (%)
T1	84.23^c^	75.32^d^	84.76^c^	9.06^a^
T2	87.35^b^	85.79^b^	93.85^a^	5.38^b^
T3	90.23^a^	87.87^a^	95.38^a^	4.73^b^
T4	85.77^bc^	83.94^c^	89.22^b^	4.80^b^
SEM	0.64	0.50	0.57	0.47
p-value	<0.01	<0.001	<0.001	<0.001

TES, total eggs set; FES, fertile eggs set; SEM, standard error of the mean.

1) T1, control group; T2, control+SSP at 0.0176%; T3, control+SSP at 0.026%; T4, control+SSP at 0.0528%.

a,b,c,d Means within a column with no common superscripts differ significantly (p<0.05).
